# Modeling Tunable Fracture in Hydrogel Shell Structures for Biomedical Applications

**DOI:** 10.3390/gels8080515

**Published:** 2022-08-18

**Authors:** Gang Zhang, Hai Qiu, Khalil I. Elkhodary, Shan Tang, Dan Peng

**Affiliations:** 1 Hubei Provincial Key Laboratory of Chemical Equipment Intensification and Intrinsic Safety, Wuhan 430205, China; 2 School of Mechanical and Electrical Engineering, Wuhan Institute of Technology, Wuhan 430200, China; 3 School of Mechanical Engineering, Jiangsu University of Science and Technology, Zhenjiang 212003, China; 4 The Department of Mechanical Engineering, The American University in Cairo, New Cairo 11835, Egypt; 5 Department of Engineering Mechanics, Dalian University of Technology, Dalian 116024, China; 6 State Key Laboratory of Structural Analysis for Industrial Equipment, International Research Center for Computational Mechanics, Department of Engineering Mechanics, Dalian University of Technology, Dalian 116024, China; 7 Department of Neurology, The Second Hospital of Dalian Medical University, Dalian 116023, China

**Keywords:** hydrogels, curved shell, biomedical devices, phase field

## Abstract

Hydrogels are nowadays widely used in various biomedical applications, and show great potential for the making of devices such as biosensors, drug- delivery vectors, carriers, or matrices for cell cultures in tissue engineering, etc. In these applications, due to the irregular complex surface of the human body or its organs/structures, the devices are often designed with a small thickness, and are required to be flexible when attached to biological surfaces. The devices will deform as driven by human motion and under external loading. In terms of mechanical modeling, most of these devices can be abstracted as shells. In this paper, we propose a mixed graph-finite element method (FEM) phase field approach to model the fracture of curved shells composed of hydrogels, for biomedical applications. We present herein examples for the fracture of a wearable biosensor, a membrane-coated drug, and a matrix for a cell culture, each made of a hydrogel. Used in combination with experimental material testing, our method opens a new pathway to the efficient modeling of fracture in biomedical devices with surfaces of arbitrary curvature, helping in the design of devices with tunable fracture properties.

## 1. Introduction

**Figure 1:**
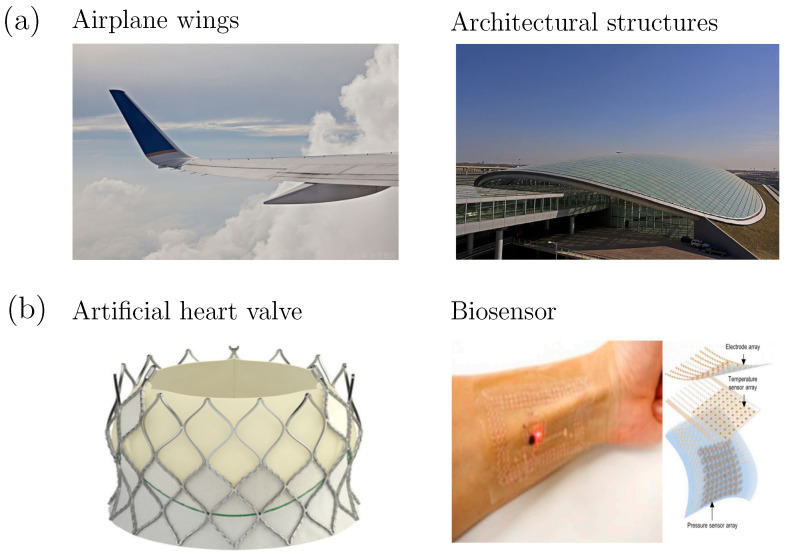
Shell structure. (**a**) Aircraft wing [18] and architectural structures [19]; (**b**) Artificial heart valve (reprinted with permission from Ref. [20]. Copyright 2017, Elsevier) and biosensor (reprinted with permission from Ref. [21]. Copyright 2020, Springer Nature).

Hydrogels are three-dimensional hydrophilic, cross-linked polymeric networks which can absorb water, or biological fluids, and swell with the water, without dissolving in it. Due to the advantages of high biocompatibility and tunable biodegradability, hydrogels are increasingly used for controllable devices in medical applications [1,2,3,4,5,6,7]. Various strategies have been developed to synthesize hydrogels. The polymeric network structure and its chemical composition can be adjusted to control the capability for water absorption, and to achieve desirable mechanical and functional properties, such as strength or conductivity. Filler materials, such as graphene, carbon nanotubes, carbon black, metallic nanowires, and nanoparticles can also be introduced to further tune their mechanical and conductive properties [8,9,10,11]. Introducing stimuli-responsive functional groups into the polymeric networks, hydrogel properties can also be controlled by environmental stimuli, including heat, light, magnetic fields, chemical agents, and acidity (pH) [12,13,14,15,16]. Due to the tunability of their material properties, hydrogels are becoming increasingly used to fabricate biomedical devices such as biosensors, capsules for drug delivery, and scaffolds for cell cultures, etc. As the human body and its organs/substructures often have irregular surfaces of complex shape, these devices are often designed with a small thickness, to better adapt to such biological surfaces. Drawing parallels to the more frequently encountered large-scale engineering structures, such as airplane wings and architectural structures, as shown in Figure 1, devices in biomedical engineering can also be considered as shell structures, in terms of their mechanical modeling, as shown in Figure 1b. This parallel is expedient as, in some biomedical applications, structural integrity of the devices should be maintained, and crack growth and propagation should be prevented, whereas in other applications cracks on a device can be harnessed to realize complex functions, such as camouflage [17]. Shell modeling of fracture may thus be called upon to cheaply and accurately explore such diverse applications for hydrogels, and to better tune their properties.

To produce devices made from hydrogels with tunable fracture properties (fracture path, pattern, and toughness), trial-and-error via experimentation may be a possible route. However, the usual mechanical tests, such as puncturing, and uniaxial tension or compression, are typically used to measure the corresponding properties prior to crack growth, such as the Young’s modulus and the fracture energy, whether directly or indirectly. Additional tests are frequently needed to further measure the fracture path, its evolving pattern, and fracture toughness. However, reproducing realistic loading and operating conditions for these devices that simulate the living body can be challenging. Furthermore, identifying the mechanisms that underly the evolving fracture may not be straightforward with such experiments. Thus, to reduce trial-and-error cost and complexity, to improve the efficiency of hydrogel development cycles, and to deepen our understanding, various computational simulations have been more recently performed. Fracture modeling of hydrogels, however, has proven challenging. Several examples are listed here for illustration of the state of the art. Mao et al. [22] developed a large deformation viscoelastic model for a double-network hydrogel, taking into account the debonding process for both polymeric networks and the swollen hydrogel. The final fracture of the double network hydrogel was not considered, however. Similarly, based on their previous work on the large deformation of nonlinear viscoelastic materials, which incorporates the kinetics of breaking physical crosslinks [23,24], Guo et al. [25] further proposed a novel time-integration scheme that investigated the stress and deformation fields near a crack tip for (single) edge-cracked specimens. Likewise, the rate-dependent fracture behavior of a polyvinyl alcohol (PVA) hydrogel was studied via experiments and numerical simulation. Liu et al. [26,27] developed a constitutive model that could accurately describe the mechanical behavior of double cross-linked hydrogels at different temperatures and loading rates. Mode-I crack tip stress fields, and the corresponding stress-intensity factors for the hydrogel were then analyzed through a finite element analysis. Shen and Vernerey [28] developed a framework to capture the interplay between energy dissipation and crack propagation in hydrogels constituted of transient networks. A cohesive zone model was then employed to relate the crack driving force to crack initiation (instability) and propagation velocity. Liu et al. [29,30] furthermore proposed a physically based poro-visco-hyperelastic model within the framework of the theory of porous media under finite strain to describe the solid-fluid-coupled material behavior of hydrogels. Their model was applied to study time-dependent fiber-reinforced hydrogel composites, and fatigue crack growth in them.

For these developed material models, it might seem straightforward to extend their modeling to shells of hydrogels. However, these models mostly focus on the underlying mechanisms of diffusion, bonding, and debonding in transient networks or double networks, and their coupling to applied deformation. They involve many material parameters, which may not be easily calibrated through experiments. Also, their programmatic implementation is relatively sophisticated. As such, they are not readily amenable for use with engineering design. Specifically for bio-device design, when its characteristic deformation time-scale is considerably smaller than the time-scale of water diffusion, the accounted-for diffusion mechanisms should be ignored [31], bringing the validity of some of these material models into question at small scales. We further note that a tensile stress state is considered primary in a typical hydrogel model. However, shell structures (curved bio-devices) may operate under severe loading conditions, where large deformation arises with shear or compression dominating the stress state. Typical hydrogel models might thus lead to unstable computations under such conditions.

It is also important to note the difficulty in modeling the entire fracture process computationally, while accounting for the path and pattern of fracture. Established methods for numerical fracture modeling in engineering design, such as the cohesive zone model and XFEM [32,33,34,35], as available today in commercial finite-element software, do not typically fair well when modeling complex fracture propagation modes, such as crack branching and merging. Moreover, elements of $C^{1}$ continuous basis functions are preferable when the modeling shells. However, most available elements in the commercial software are $C^{0}$.

It seems to us that in view of these many challenges, studies that model fracture in shells composed of hydrogels, especially for biomedical applications, are today rare. We thus aim to model the large deformation of hydrogel shells of arbitrary curvature using $C^{0}$ elements, as available in finite-element software, with application to bio-devices. In this paper, we adopt the phase-field model, which renders it possible to track crack initiation and propagation with greater ease. Phase field can be discretized via the graph method, which is widely used in the computational geometry, machine learning and computer science communities [36,37,38]. For hydrogels, we consider a nonlinear elastic model, in which the deformation energy is decomposed into tensile and compressive parts. This decomposition can enhance numerical stability when $C^{0}$ solid shell elements are used. Although the proposed model is phenomenological, it can relate to water content, the fillers, and the stimuli, by means of experimental data. This model aims to be is easier and more convenient for users who are not familiar with the relevant mathematics and theories in mechanics. It is consistent with the idea of data-driven computational mechanics, which has been developing very rapidly in recent years [39,40,41,42,43,44,45,46,47]. In Section 2, this material model with phase field degradation is presented. The phase field is discretized based on the graph method. In Section 3, a staggered solution method is proposed. We then model the fracture of a wearable biosensor, a membrane-coated drug, and a matrix for a cell culture, as illustrative examples that highlight the strength of our proposed method. Finally, we summarize our main results and conclude our work in Section 4.

## 2. Material Models for Fracture

To model the deformation and fracture of hydrogels, a phenomenological mechanical model with few parameters is proposed. To help preserve numerical stability, deformation is decomposed into tensile and compressive parts. We first define $\lambda_{i}$ as the *i*-th principal stretch of the deformation gradient $F_{iJ}$. The first invariant of the right Cauchy–Green strain is $I_{1}=\sum_{i=1}^{3}\lambda_{i}^{2}$, and the Jacobian of the deformation gradient is $J=\lambda_{1}\lambda_{2}\lambda_{3}$. The phase field *p* can be introduced to the free energy density *W* per the following,

(1) $$W=\left[\left(1-K\right)p^{2}+K\right]W^{+}+W^{-},$$

to model the hyperelastic deformation of hydrogels with damage. Our model assumes that crack growth occurs faster than the rate of water diffusion in the hydrogels, so that their viscous behavior can be ignored [48,49]. For readers who are not familiar with phase field fracture modeling, the phase field *p* can be thought of as the damage variable. When it is set equal to 1, it signifies that the material has full integrity, and no damage. When it is set equal to zero, it signifies that the material is in a fully damaged state. K is a numerical constant that is correspondingly often set to a small value to enhance numerical stability, as was shown for the small deformation regime [50,51,52,53] and the large deformation regime [48,54]. $W^{+}$ and $W^{-}$ are defined by

$$W^{+}=G\left(\lambda_{i}^{+},J^{+}\right)\;\mathrm{where}\;\lambda_{i}^{+}=\left\{\begin{array}{cc} \lambda_{i} & \mathrm{for}\;\lambda_{i}>1\\ 1 & \mathrm{for}\;\lambda_{i}\leq 1 \end{array}\right.\;J^{+}=\left\{\begin{array}{cc} J & \mathrm{If}\;J>1\\ 1 & \mathrm{If}\;J\leq 1 \end{array}\right.\;$$

and

$$W^{-}=G\left(\lambda_{i}^{-},J^{-}\right)\;\mathrm{where}\;\lambda_{i}^{-}=\left\{\begin{array}{cc} \lambda_{i} & \mathrm{for}\;\lambda_{i}<1\\ 1 & \mathrm{for}\;\lambda_{i}\geq 1 \end{array}\right.\;J^{-}=\left\{\begin{array}{cc} J & \mathrm{If}\;J<1\\ 1 & \mathrm{If}\;J\geq 1 \end{array}\right.\;.$$

The free energy density function *G* takes the form

(2) $$G\left(\lambda_{i},J\right)=\frac{1}{2}\mu \underset{i=1}{\sum^{3}}\left(\lambda_{i}^{2}-1-2\ln\lambda_{i}\right)+\frac{1}{2}\chi {\left(J-1\right)}^{2},$$

where μ and χ are the (initial) shear and Lamé constants, respectively. This is the neo-Hookean model, which is widely used to model soft solids, including hydrogels [55,56,57]. The first Piola–Kirchhoff (PK) stress can then be computed from

(3) $$P_{iJ}=\frac{\partial W}{\partial F_{iJ}}=\left[\left(1-K\right)p^{2}+K\right]\frac{\partial W^{+}}{\partial F_{iJ}}+\frac{\partial W^{-}}{\partial F_{iJ}}.$$

### 2.1. Phase Field Discretization

The evolution of the phase field identifies the fracture process. A Lagrangian formulation is adopted to discretize the phase field equations. All integrations are performed on the undeformed configuration, and differentiation is computed with respect to its reference coordinates. We also assume, for simplicity of computational implementation, that the phase field variable at each Gaussian integration point will take the same value within an element (usually more than three Gaussian points are considered along the thickness direction of a shell element), which greatly reduces computational cost. Specifically, as the fracture process evolves, only a single phase field value for every element in the mesh is computed and stored, which is interpreted as an average phase field value for all the Gaussian points in that element. This assumption has been successfully employed by [58]. Consistent with this assumption, the energy density can be therefore averaged over the element’s thickness,

$${\bar{W}}^{+}=\frac{1}{N_{G}}\sum_{I=1}^{N_{G}}{\bar{W}}_{I}^{+},$$

where ${\bar{W}}_{I}^{+}$ is the energy density at the *I*-th Gaussian point, and $N_{G}$ is the number of Gaussian points along the thickness direction of a shell element.

Now, a correspondence between a finite element mesh and a graph may be easily established. The finite element mesh for a curved surface can be considered as a weighted, undirected graph. A graph is here defined as the pair $\left(V,E\right)$ where *V* denotes the set of *n* vertices (a vertex is equivalent to a node in finite-element terminology), and E⊆V×V the set of edges. Therefore, the cotangent formulation from discrete differential geometry can be leveraged to obtain the discrete phase field equations. At a vertex point *i*, this can be written as

(4) $$\left[4\ell \left(1-K\right){\bar{W}}^{+}/G_{c}+1\right]p_{i}+4\ell^{2}\frac{1}{a_{i}}\sum_{j:\left(i,j\right)\in E}w_{ij}\left(p_{i}-p_{j}\right)=1.$$

Note that *i*, *j* label the finite-element node numbers. $\left(i,j\right)\in E$ represents an edge in the graph. *ℓ* is the length scale parameter in the phase field model, which usually characterizes the width of a crack. $a_{i}$ and $w_{ij}$ are related to the area and the length of the element on the graph. $G_{c}$ is correspondingly the fracture energy per unit area for an element. For the detailed derivation process, see Section 3 of Zhang et al. [54].

As we discussed in the introduction, many mathematical material models have been already proposed in the literature [22,23,24,25,26,27,28,29,30] to model the fracture of the gels. However, most models seem significantly more complex than what we propose herein. Our model for gels only requires three main parameters (Young’s modulus, bulk modulus, and the fracture energy per unit area). The corresponding polymer structures that relate to the three parameters can be tuned to control the fracture pattern and the toughness of the bio-devices made by the gels discussed further in Section 2.3.

Finite-element modeling of the fracture of plates and shells has also attracted much research interest for a long time, with discrete and diffuse methods having been proposed. In discrete methods, cracks are taken as explicit discontinuities in the displacement field. Typical methods are the extended finite element method (XFEM) and the cohesive zone method, first proposed by [32,35,59]. It is difficult for these models to simulate complex fracture modes, such as crack branching and merging. In diffuse fracture methods, cracks are smeared out and represented directly by a damage variable in the constitutive formulation. A popular diffuse damage model is the phase-field approach [48,54,60,61]. However, discretizing a phase field in thin shells by using modern discrete differential geometry, as we pursued herein, is rare for fracture modeling. Specifically, our discretization process preserves the governing differential equations’ intrinsic geometric structure when on non-flat manifolds (shells). Convergence is thus guaranteed even with $C^{0}$ continuity elements [62], which is a difficulty for traditional finite-element modeling. We thus showed how to employ our resulting modeling tool to accurately capture the fracture of bio-devices made of gels.

### 2.2. A Material Model for Heart Tissue

In addition to modeling the hydrogel, a bio-device may be for instance implanted on the heart’s surface, as will be modeled in a later example. A model for cardiac tissue is thus also required. The Ogden model [63,64] is herein employed. The Ogden model shows great applicability to nonlinear elastic materials, including soft tissue, and retains a simple form. The form of the free energy density for the Odgen model is given by

(5) $$W=\frac{2\mu_{Og}}{\alpha^{2}}\left({\bar{\lambda }}_{1}^{\alpha }+{\bar{\lambda }}_{2}^{\alpha }+{\bar{\lambda }}_{3}^{\alpha }-3\right)+\frac{1}{2}K_{Og}{\left(J-1\right)}^{2},$$

where ${\bar{\lambda }}_{i}\left(i=1\cdot \cdot \cdot 3\right)$ is the deviatoric principal stretch, which is defined as ${\bar{\lambda }}_{i}=J^{-1/3}\lambda_{i}$. $\mu_{Og}$ and $K_{Og}$ are the shear modulus and the bulk modulus respectively, and α is a material constant. The first PK stress for the Odgen model can be computed from

(6) $$P_{iJ}=\frac{\partial W}{\partial F_{iJ}},$$

similar to our model for the hydrogels.

Three material models (e.g., Mooney–Rivlin, Yeoh, Ogden model) are often used to describe the mechanical response of heart tissue. The Mooney–Rivlin model is only suitable for small deformation and cannot describe well the mechanical behavior of heart tissue under multiaxial loadings, ignoring the effect of fiber orientation. The Yeoh model is a special form of reduced-polynomial hyperelastic model, which can describe well the mechanical behavior of tissue during tensile and shear deformation. However, too many material parameters are required. Conversely, the Ogden model is expressed by elongation in three principal directions, using few parameters, and can be effectively and efficiently used to model the mechanical behavior of heart tissue [65,66].

### 2.3. Toward Tuned Fracture Properties of Devices

**Figure 2:**
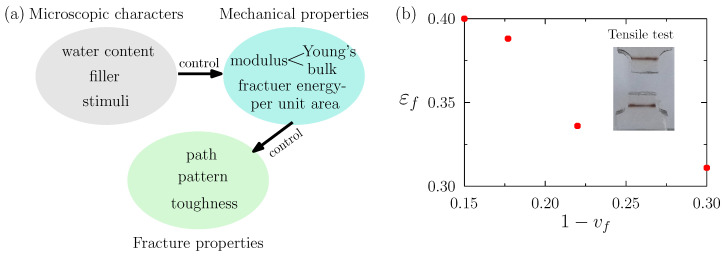
(**a**) Tuning the fracture property by microscopic characters such as water content, filler or stimuli for hydrogels. (**b**) Results of tensile fracture test for a brittle hydrogel (fracture strain vs. water volume fraction).

As discussed in our introduction, and summarized in Figure 2a, the water content, the content and distribution of fillers (e.g., graphene, carbon nanotubes, carbon black, metallic nanowires and nanoparticles), and the stimuli (heat, light, magnetic fields, chemical agents, and pH) tune the mechanical and fracture properties of hydrogels. In terms of mechanical modeling, careful constitutive models for each hydrogel in each situation should be built, if we wish to model the hydrogel accurately. However, these are not easy to build. Many such material models are complicated by their attempted coverage of the underlying mechanisms of deformation [22,23,24,25,26,27,28]. This complication leads to a plurality of material parameters that are often difficult to calibrate. Such in-depth modeling is not amenable for detailed fracture modeling (itself complex), limiting its scope of use in device design. Indeed, we developed in earlier work a material model for hydrogels that considers the contained water and its interaction with the polymeric network. We used that in-depth model to simulate the fracture of a hydrogel block under compressive loading, and found that its predicted fracture pattern is less accurate than that obtained by the phenomenological model that we propose in the present paper, when compared against experiments [48,54,67].

Our proposed material model herein is also easier for implementation and computation. In fact, water content, content and distribution of fillers, and environmental stimuli, can all be accounted for in the material’s Young’s modulus *E*, bulk modulus *K*, and fracture energy per unit area $G_{c}$.

Here, we consider an example. We fabricated in our labs a poly-acrylamide hydrogel for tensile testing. The hydrogel, with different initial volume fractions of water ($v_{f}$), is prepared by first loading the glass container with an aqueous pre-gel solution and N,N’-methylene-bis-acrylamide. Gelation can be initiated by adding an ammonium persulfate aqueous solution as the initiator, and N,N,N’,N’-tetramethylenediamine as the catalyst, into the pre-gel solution. In our previous work, we have demonstrated the thermal stability and chemical stability of this fabricated gel, which we used to study experimentally the phenomena of surface morphology transition and fracture [48,68]. That is, we showed that gel structure and properties do not change significantly for a wide range of temperatures and under the action of its chemical environment. The fabrication method is reproducible, which are used in several our previous works [48,68]. In this paper, we do not try to show that our fabrication method is advantageous over other methods. We just take the fabricated gel as an example to demonstrate that the relevant mechanical parameters of the gel can be measured. With these measured parameters, it is possible for us to accurately simulate the fracture behavior of the hydrogel in biomedical applications and conveniently control the fracture, show in the numerical results section.

Via this method, three different hydrogels with different initial volume fractions of water are fabricated. Tensile testing until fracture is then carried out. The results are presented in Figure 2b. It can be seen that for an increasing initial volume fraction of water, the fracture strain is much larger. This finding implies that the fracture energy per unit area $G_{c}$ increases with water content. This calibrated $G_{c}$ can be therefore used to model the fracture process of a hydrogel device via our proposed computational framework. Finally, the water content can be altered when fabricating, to tune the fracture property of the device, based on the simulation results obtained. As such, if experiments are performed that vary the water content, the content and distribution of the filler, and the stimuli, one can with ease connect experimental data to the Young’s modulus *E*, bulk modulus *K*, and/or the fracture energy per unit area $G_{c}$ in our computational model for device design. There are also many ways to build this relationship through machine learning [69,70,71]. Therefore, our method can supplement and guide experiments to more easily tune the desired fracture properties of bio-devices.

The present work focused on the tunable fracture of bio-devices made of gels. Indeed, tunable hydrogels and their applications are a hot research area. A tunable hydrogel can be important in a variety of applications, including wound healing, hemostasis, drug delivery, cell encapsulation and release, 3D bioprinting, and tissue engineering. For example, Bratskaya et al. [72] discussed the applicability of a stimuli-responsive hydrogel of tunable dissolution rate under physiological conditions. Similarly, Danko et al. [73] prepared a polyelectrolyte hydrogel, the swelling and water state of which can be controlled by external stimulation. Their hydrogel shows a capability of free-floating on the water surface, indicating its potential use in floating pH detection devices. In the same vein, Jafari et al. [74] developed hydrogels of controllable gelation time and adjustable degradation rate. Their new hydrogel exhibited remarkable prospects for the reconstruction of artificial ovaries. However, most of these tunable gel properties are oriented toward the functionality of the hydrogels. At present, there are too few studies on tunable gel fracture in bio-devices, risking the reliability of these otherwise impressive advances.

## 3. Numerical Results

We apply a staggered scheme to solve the evolutionary equations of fracture, leveraging the finite-element method. In this scheme, at a given time step, the mechanical equilibrium equation is solved first to update the displacement. With the updated displacements, the phase field equation on all the nodes is then solved. Once both the displacement and the phase fields have converged, the computation will then proceed to the next time step. Thus, the original coupled problem is reduced to two smaller systems, which renders the algorithm more numerically stable, as per our numerical simulations.

To demonstrate the capabilities of our proposed approach for hydrogel device design in medical applications, the fracture modeling of a wearable biosensor, a membrane-coated drug, and a matrix for a cell culture, is carried out. We adopt nondimensional units in all our simulations.

### 3.1. A Wearable Biosensor

In vivo monitoring of heath is very important for many diseases. Biosensors with programmed diagnostic functions can measure, for instance, blood temperature, pressure, pH, glucose concentration, hormonal, and alcohol levels, and are becoming more and more popular. They can also can be used to detect human movement, or small toxic molecules [75,76,77,78]. Biosensors usually attach to specified positions in the human body, where important biomarkers (e.g., pressure, alcohol level) can be best collected. They can be attached externally (e.g., on the skin) or internally (e.g., on the surface of heart). Hydrogels have been taken as excellent candidates for such applications, specifically due to their biocompatibility, mechanical flexibility, and biodegradability. Such devices should be designed with good mechanical strength to resist fracture, and to maintain robust adhesion onto dynamic, curved surfaces (such as the skin, the heart, etc.).

**Figure 3:**
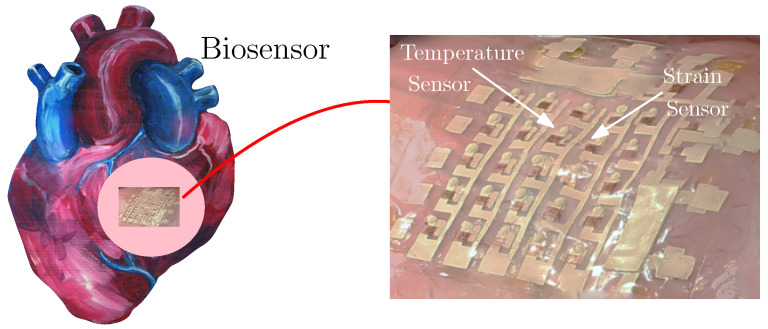
Schematic diagram of biosensor monitoring heart health [80].

Let us here consider the example of a biosensor for the heart, as shown in Figure 3 [79]. Note how the heart surface is curved and irregular (of varying curvature). The biosensor must be attached to this surface, which expands and contracts non-uniformly as the heart beats. How to ensure the integrity and the cohesive strength of the biosensor on this dynamic surface is a very important engineering design problem. An accurate evaluation of its integrity and bonding strength could help tune the functionality and longevity of the device, to minimize patient exposure to additional surgery.

**Figure 4:**
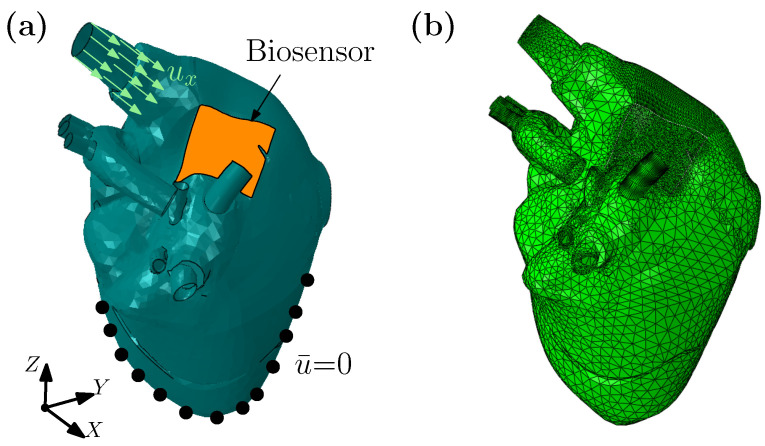
Fracture of a biosensor attached to the heart. (**a**) Geometric dimensions and boundary conditions of a heart model. (**b**) Mesh model of heart.

To study fracture initiation and propagation in such biosensors, we establish two shell models, one for the heart, and another for the biosensor. Both the heart model and biosensor model are meshed, using a finite element mesher. The heart model contains 14,485 triangular shell elements, and 7594 nodes, with a finer mesh used in the area where it comes into contact with the biosensor. The mesh and coordinates are shown (see Figure 4a). An Odgen model is used to describe the mechanical response. Similarly, the biosensor contains 5242 triangular shell elements and 2732 nodes, and are attached to the heart model through a tie-binding contact technology [81]. Our phase field model is employed to describe the biosensor’s mechanical behavior. The shear modulus and the Lamé constants of the biosensor model are taken as, μ=1.0 and χ/μ=2.17, respectively. The diffuse length scale (for the crack width) in our phase field is set at ℓ=1.0, and the fracture energy density is varied between $G_{c}/\left(\mu \ell \right)=0.3\times 10^{-4}$, $0.6\times 10^{-4}$ and $1.2\times 10^{-4}$, respectively. The effective element size in the critical zone is $h_{ele}/\ell \approx 0.1$, which is less than *ℓ*. The elastic parameters for the Odgen material model are $\mu_{Og}/\mu$ = 0.1, $K_{Og}/\mu =1.0$, and α = −20.0 [82]. The displacement along the radial direction is imposed at the wall of the large blood vessel, and the bottom part (apex to midline, as marked by black points) is fixed, as shown in Figure 4b. This boundary condition aims to only generate physiologically consistent maximum stretches in the atrium’s tissue beneath the biosensor, in a given cardiac cycle.

**Figure 5:**
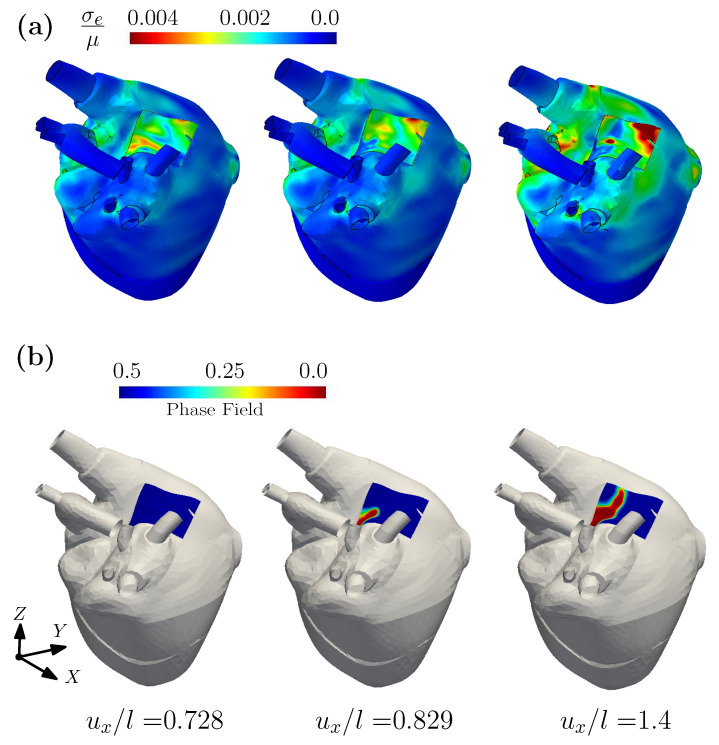
(**a**) The contour plots of normalized effective Cauchy stress and (**b**) the contour plots of phase field for a heart model and a biosensor shell, under different displacement.

**Figure 6:**
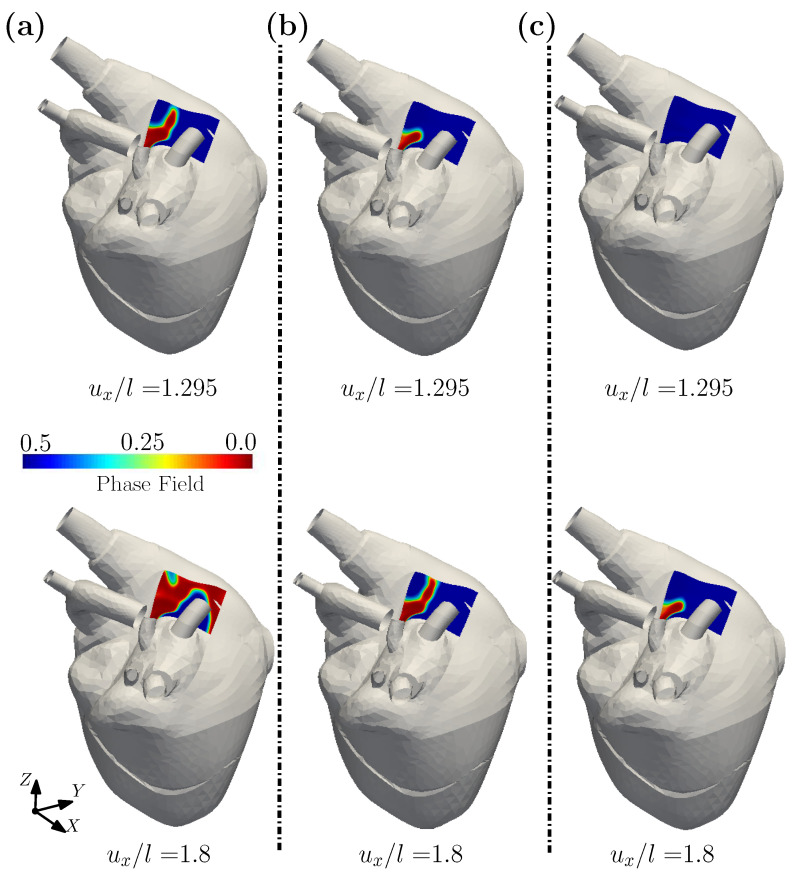
The contour plots of phase field for a biosensor shell with different $G_{c}/\left(\mu \ell \right)$ = (**a**) $0.3\times 10^{-4}$; (**b**) $0.6\times 10^{-4}$; (**c**) $1.2\times 10^{-4}$.

Figure 5 plots the contours of the effective Cauchy stress and the phase fields for $G_{c}/\left(\mu \ell \right)=0.3\times 10^{-4}$, as generated under three levels of imposed displacement $u_{x}/l$. For an imposed displacement $u_{x}/l$ = 0.728, the fracture phase field is essentially undeveloped (see Figure 5b). When $u_{x}/l$ reaches 0.829, the fracture phase field begins to develop at the left corner of the biosensor. With increasing displacement, the crack propagates until full fracture of the biosensor. Figure 6 plots the contours of the phase field variable for $G_{c}/\left(\mu \ell \right)=0.3\times 10^{-4}$, $0.6\times 10^{-4}$ and $1.2\times 10^{-4}$, respectively. From the Figure 6, it can be seen that due to the different values of $G_{c}/\left(\mu \ell \right)$, the damage modes in the biosensor’s shell are different for the same displacement. With an increase to $G_{c}$, the imposed displacement required for crack initiation, and for complete fracture, will also increase. When designing these sensors, an appropriate $G_{c}$ may be obtained by tuning the water content or the volume fraction of the filler, which can prevent the biosensor from the premature damage during the operational life of the biosensor.

In summary, wearable flexible sensors [83,84,85] for human health monitoring [86,87,88,89,90] have shown new advances. These flexible sensors can better monitor subtle changes in the external environment, such as temperature [91,92,93], humidity [91,94], and deformation [95,96], and are evolving rather fast. For these kinds of sensors, various types of hydrogels are being actively developed, such as cellulose hydrogels [97], PNIPAM hydrogels [98], and double chain hydrogels [99]. These hydrogels are sensitive to mechanical signals and can be used as dynamic soft sensors that accurately detect small external forces and strains. However, it should be noted that there are too few computational studies to ensure the good mechanical strength of the resulting hydrogel devices, their ability to resist fracture, or their robust adhesion onto dynamic, deformable, curved surfaces (such as the skin, the heart, etc.). Our approach herein aims to serve as a first sure step in filling this knowledge gap.

### 3.2. Outer Membrane of Drug-Delivery Capsule

Hydrogels have received a great deal of research attention in the field of drug delivery. Indeed, there is much literature and many patents on the application of hydrogels to drug delivery [4,100,101]. Hydrogels usually have a high porosity, which can be controlled by the cross-linkers in the polymeric network, and its affinity to water. The porous structure allows the drug to be first loaded, then gradually released at a controlled rate, for sustained periods. The release of the drug can in fact be controlled rather flexibly by tuning the diffusion, swelling, chemical potential, or the environment (such as PH).

**Figure 7:**
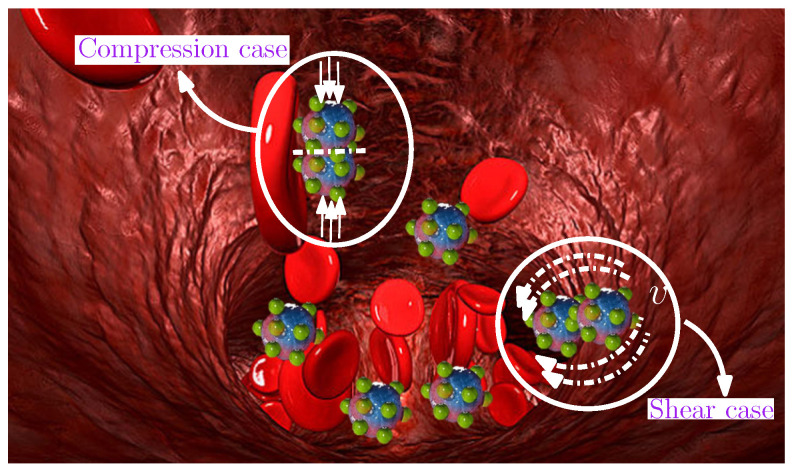
Schematic diagram of drug delivery in blood vessels [102].

A diffusion-controlled drug release system can be represented by a reservoir delivery system, which is usually composed of a drug-containing core, coated with a hydrogel membrane, as with commonly available capsules (cylinders, slabs, or spheres). When this reservoir system is transported in the blood vessel, it is subjected to interaction with a dynamic fluid (blood), with compression and shear acting on the capsule’s membrane, as shown in Figure 7. Although the porous hydrogel brings advantages to drug release, nevertheless, the same voids induce a lower mechanical strength, which may undermine the capsule’s integrity and sustained functionality.

**Figure 8:**
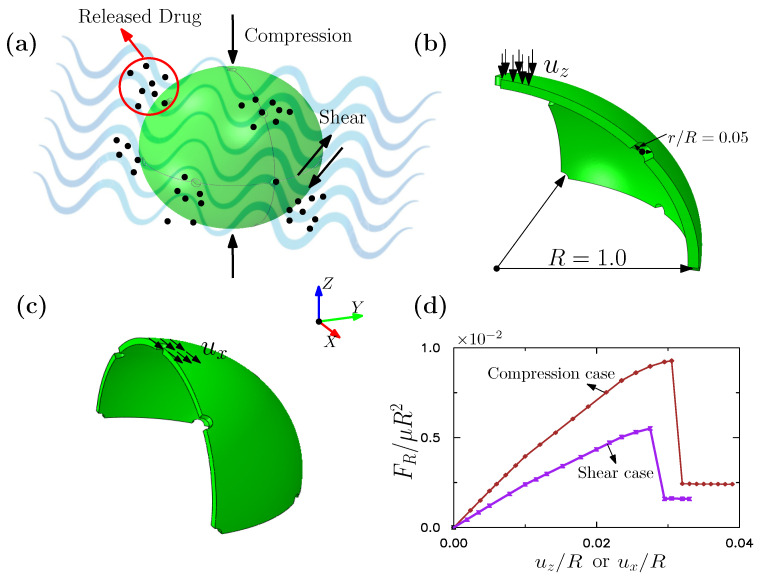
Fracture of a spherical shell subjected to compression and shear. (**a**) Spherical shell model for drug delivery. (**b**) One eighth spherical shell model. (**c**) A quarter spherical shell model. Geometric dimensions and boundary conditions of the model are given. (**d**) The reaction force vs. displacement curves.

We here examine the fracture of a spherical drug-delivery capsule. A spherical shell model is built with a radius R=1.0, which contains multiple holes of radius r/R=0.05 that facilitate the release of drugs. The holes are uniformly distributed on the surface of the sphere, as shown in Figure 8a. In this study, we consider two external loading conditions, as shown in Figure 8b,c respectively. The two loadings approximately consider the compression and shear forces acting on the capsule’s membrane from the dynamic fluids in which the capsule is immersed. Considering the symmetry of the spherical shell and the imposed loading, only a quarter and an eighth of the full sphere are here analyzed, for the shear and compression-loading scenarios respectively, as noted in Figure 8b,c. The shear modulus and the Lamé constants of the membrane are μ=1.0 and χ/μ=2.17, respectively. For the compression case, the model contains 11,108 triangular shell elements and 5700 nodes. For the shear case, the model contains 12,456 triangular shell elements and 6428 nodes. The two cases have the same shell radius, shell thickness, length scale *ℓ*, and fracture energy per unit area $G_{c}$. The length scale for the phase field model is set at ℓ/R=0.06, and the fracture energy per unit area is taken as $G_{c}/\left(\mu R\right)=0.3\times 10^{-3}$. The effective element size in the critical zone is less than *ℓ*. Figure 8d shows the normalized resultant reaction force vs. the imposed displacement for two cases, where brown and purple represent the compression case and the shear case respectively. It can be seen that the resultant force increases linearly at the beginning, followed by a softening phase, then suddenly dropping to a very low value with fracture.

**Figure 9:**
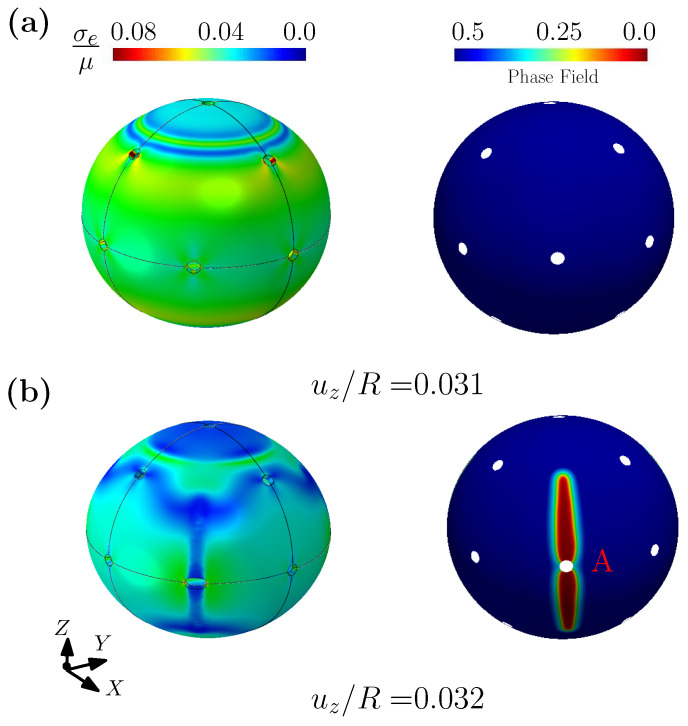
The contour plots of normalized effective Cauchy stress and phase field for spherical shell considering compression, under different displacement $u_{z}/R$ =: (**a**) 0.031; (**b**) 0.032.

**Figure 10:**
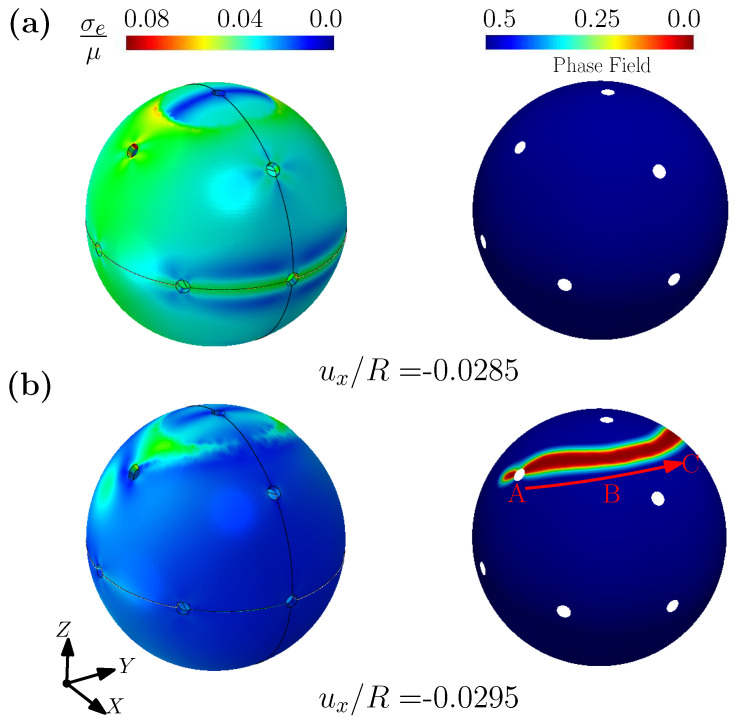
The contour plots of normalized effective Cauchy stress and phase field for spherical shell considering shear, under different displacement $u_{x}/R$ =: (**a**) −0.0285; (**b**) −0.0295.

Figure 9 shows the contours of effective stress and phase field, at different levels of imposed displacement for the compression case. The left column of the figure plots contours of the effective stress, while the right column of the figure shows the contours of the fracture phase field. At the imposed displacement of $u_{z}/R$ = 0.031, the spherical shell is deformed, but the fracture phase field has not yet developed. At an imposed displacement $u_{z}/R$ = 0.032, it can be seen that the fracture phase field begins to develop around hole A (marked in the figure) and rapidly expands along the Z direction (see Figure 9b). It should be noted that due to the symmetry of the shell, there is also a corresponding fracture phase field at a symmetrical position on the sphere (not shown in the figure). Similar to Figure 9, Figure 10 shows the contours of effective stress and fracture phase field for the case of shear. Different from the compression case, when the fracture phase field appears around point A it extends along a direction towards point B and point C (point A, B and C are marked in Figure 10b). Finally, noting that the velocity of blood varies with the different blood vessel diameters in the body, we can expect different loading conditions and force distributions on the capsule’s surface (combinations between compression and shear). Our simulation thus shows that using hydrogels for the membrane of a capsules should be carefully designed to resist fracture under different external loading conditions, and may differ depending on the target organ and the hemodynamic pathway followed.

It can be summarized that hydrogels have broad application prospects for controllable drug delivery, by virtue of their unique programmability, designable responsiveness, biocompatibility, and biodegradability. These desirable properties can offset prevalent shortcomings in traditional drug-delivery materials, such as their high invasiveness, low efficiency, and intermittent local delivery [103,104]. To further promote the application of hydrogels in drug delivery, DNA hydrogels [105], and smart hydrogels [106] have also been recently developed. However, hydrogels are prone to fracture when interacting with the dynamic blood flow during the process of drug delivery [29,107,108], decreasing their success probability at the target. Our proposed method can capture this fracturing process during drug delivery so that the hydrogel device can be designed for improved success rates.

### 3.3. A Fibered Matrix for a Cell Culture

Hydrogel usage as scaffolds requires it to combine with biological cells to generate living tissue, both in vitro and in vivo. Such construction of three-dimensional regenerative tissue further requires that we create an environment that enables the biological cells to grow, and to interact with their surroundings. The porous microstructure of the hydrogels is critical for such cell growth. The matrix for a cell culture is often designed in the form of thin films composed of nanofibers or nanosheets. Again, the voids on these films often result in lowered mechanical strength, which can compromise scaffold functionality.

**Figure 11:**
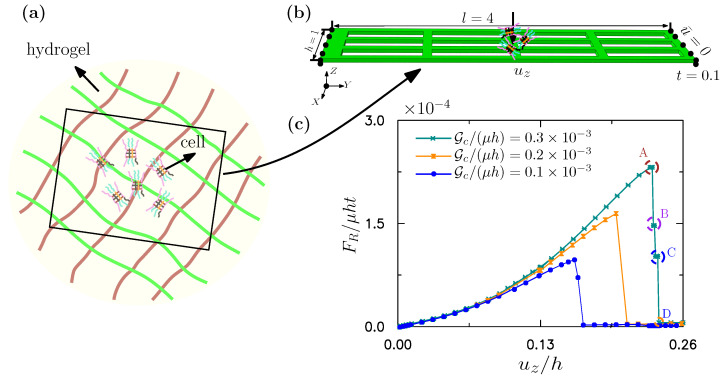
Fracture of hydrogel matrix for cell culture. (**a**) Schematics of cell culture on the hydrogel matrix. (**b**) Geometric dimensions and boundary conditions of the simplified model. (**c**) The normalized reaction force vs. the normalized displacement.

The fracture of porous sheets is here examined. A sheet can be modeled as a shell. A fibered topology is here taken as an example, as shown in Figure 11a. It can be fabricated by tetrazine-norbornene chemistry [109]. A finite-element model for this fibered shell structure has been here developed. The size and boundary conditions of the model are shown in Figure 11b. The length *l*, width *h*, and thickness *t* of the model are 4.0, 1.0 and 0.1, respectively. The mesh includes 10,330 triangular shell elements and 6032 nodes. The left and right edges of the model are fixed, and a displacement along the *z* direction is imposed at the middle of the model, which mimics the weight of a cell on the hydrogel. The shear modulus and Lamé constants of the fibered structure are taken as μ=1.0 and χ/μ=2.17, respectively. The length scale is set at ℓ/h=0.06, and the fracture energy per unit area is varied between $G_{c}/\left(\mu h\right)=0.1\times 10^{-3}$, $0.2\times 10^{-3}$ and $0.3\times 10^{-3}$, respectively. The effective element size in the critical zone is less than *ℓ*.

Figure 11c shows the reaction force $F_{R}$ along the *z* direction as a function of displacement $u_{z}$. It can be seen from Figure 11c that the reaction force first increases, then suddenly drops. We here mark four points on the unloading curve of the fracture process. Point A indicates that the shell structure has just commenced to fracture, point B and C indicate increasing damage to the shell, prior to final fracture. The reaction force then drops to its lowest value (point D) when full damage to the shell structure has occurred.

**Figure 12:**
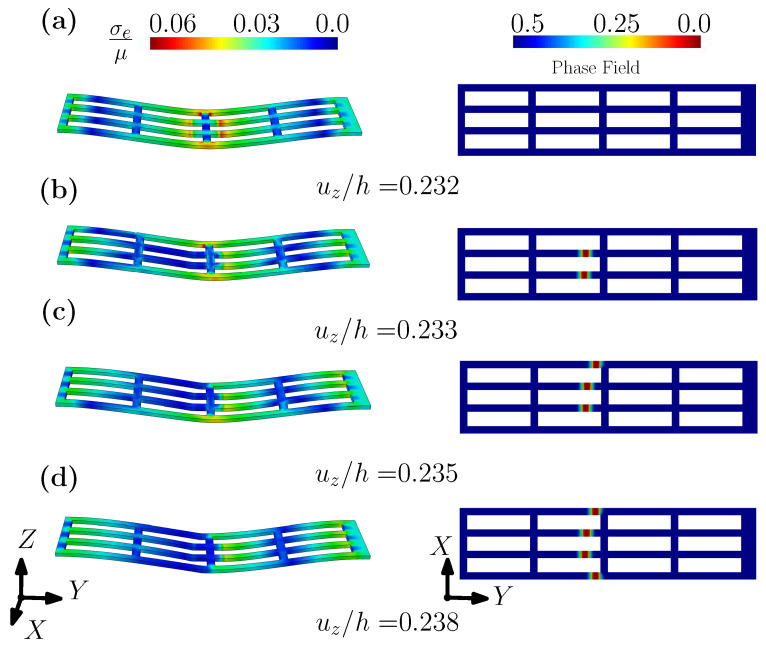
The contour plots of normalized effective Cauchy stress and phase field under different displacement $u_{z}/h$ =: (**a**) 0.232; (**b**) 0.233; (**c**) 0.235; (**d**) 0.238.

**Figure 13:**
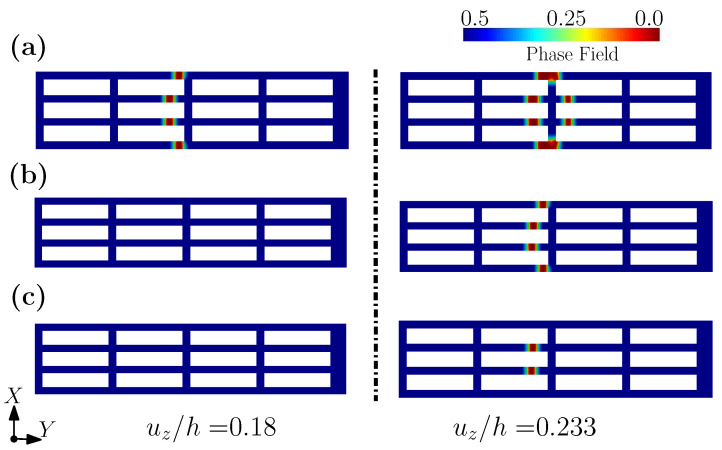
The contour plots of phase field with different $G_{c}/\left(\mu h\right)$ =: (**a**) $0.1\times 10^{-3}$; (**b**) $0.2\times 10^{-3}$; (**c**) $0.3\times 10^{-3}$.

Figure 12 plots the contours of effective stress and fracture phase fields for $G_{c}/\left(\mu h\right)=0.3\times 10^{-3}$, under different levels of imposed displacement, viz. $u_{z}/h$ = 0.232, 0.233, 0.235, and 0.238. For an imposed displacement of $u_{z}/h$ = 0.232, the shell structure has undergone large deformation, but the fracture phase field is yet undeveloped (see Figure 12a). When the applied displacement reaches $u_{z}/h=0.0233$, the fracture phase field begins to develop, which is consistent with the concentration of the effective stress, as shown in Figure 12b. With an increase to the imposed displacement, the number of the locations where the fracture initiates increases gradually, until final fracture has occurred. In addition, Figure 13 plots the contour of the fracture phase fields for three different levels of $G_{c}/\left(\mu h\right)=0.1\times 10^{-3}$, $0.2\times 10^{-3}$ and $0.3\times 10^{-3}$, under the same displacement. It can be seen from Figure 13 that due to the different values of $G_{c}/\left(\mu h\right)$, the fracture modes of the fibered structure are different under the same level of displacement. With an increase to $G_{c}$, fracture occurs only in the two fibers at the center. This example clearly shows that the fracture pattern can be controlled by $G_{c}$, which can again be achieved by tuning the hydrogel’s water content, etc.

The use of hydrogels as scaffolds has made further progress recently. For example, Liu et al. [110] prepared a thermosensitive hydrogel and used it for in situ embedding and three-dimensional culture of MCF-7 breast cancer cells. It was shown that this thermosensitive hydrogel can be used as a three-dimensional platform with high biocompatibility and easy functionalization, and has great potential in the biomedical field. Similarly, Jiao et al. [111] prepared sodium alginate-based composite hydrogels with good cell compatibility, which can be used for three-dimensional cell culture in vitro. Moreover, Nguyen et al. [112] developed a co-culture system of ECM degradation products and MSCs with macrophages. Bone marrow mesenchymal stem cells cultured on this hydrogel show immunomodulatory properties and can be used in regenerative medicine. It was shown that the hydrogel cell culture offers great prospects for the development of modern medicine. We deem that the numerical simulations of this section for hydrogel scaffolds indicate that our proposed method can be used to investigate the fracture (durability) of hydrogel scaffolds in new similar applications.

## 4. Concluding Remarks

In recent years, research into hydrogels has made significant breakthroughs (e.g., self-healing hydrogels [88,89,90,113], smart hydrogels [98], cellulose hydrogels [97], double chain hydrogels [114], supramolecular hydrogels [115], etc.). However, most of the research focuses on hydrogel functionality, as for flexible wearable sensors, drug delivery, and medical engineering. The mechanical strength of most hydrogels today, however, is still far from optimal, being easy to damage (e.g., by accidental fracture, or premature degradation), as a result of undesirable or uncontrolled growth to their micro- or macrocracks [116]. Hydrogels have various other shortcomings to be overcome by researchers, such as their easy loss of sensor signal, poor antifreeze performance at low temperature, foreign body reaction, and undesired performance losses. At present, most functional hydrogels remain in the experimental stage and have not been put into use due to their immature production technologies. Much research work is thus still needed to promote functional hydrogels in our daily lives, realize their commercialization, and translate their fullest application potential. Mechanical modeling as we propose herein can serve an important role in this pathway of their development, in particular for bio-devices.

Hydrogels, with easily tuning properties, have been widely explored as prime candidates for modern medical devices, such as biosensors, drug delivery systems, and tissue engineering applications. Because it is today challenging to model the fracture of hydrogels, there are today few computational design tools that can investigate and control the fracture behavior of their medical devices, for instance fracture initiation, fracture path and propagation pattern, and resulting toughness. In this paper, a modeling framework is laid to examine the fracture of such devices across various biomedical applications, while leveraging simple phenomenological constitutive formulations. In our approach, the devices are treated as shell structures. Then, a phase field approach is adopted to model fracture initiation and propagation in those devices, while leveraging finite-element software. By harnessing discrete differential geometry, $C^{0}$ element in the finite-element simulations can be effectively used to obtain accurate results. Moreover, as the devices are treated as shells, computational cost is greatly reduced.

Three examples of fracture modeling have been here presented; a wearable biosensor, a membrane-coated drug, and a fibered matrix for a cell culture, all composed of hydrogels. In all these examples, by tuning the fracture energy per unit area $G_{c}$, we can control fracture initiation location, and the pattern of cracking. As we showed with a simple experiment in our lab, $G_{c}$ is strongly related to water content, as well as to fillers and environmental stimuli, offering a clear avenue to tune hydrogel devices computationally. This connection of medical device performance to water content, filler choice, or stimuli should thus be carried out in future research. We also plan to collaborate with more experts on experiments that can help us solve these challenging design issues. 

## Data Availability

Not applicable.
